# The Variability of Sleep Among Elite Athletes

**DOI:** 10.1186/s40798-018-0151-2

**Published:** 2018-07-27

**Authors:** Mathieu Nedelec, Anis Aloulou, François Duforez, Tim Meyer, Gregory Dupont

**Affiliations:** 1 French Institute of Sport (INSEP), Research Department, Laboratory Sport, Expertise and Performance (EA 7370), 11 Avenue du Tremblay, 75012 Paris, France; 2 Centre du Sommeil, Hotel Dieu de Paris, 1 Place du Parvis Notre Dame, 75004 Paris, France; 3 Institute of Sport and Preventive Medicine, Saarland University, GEB. B82, 66123 Saarbrucken, Germany

**Keywords:** Recovery, Performance, Competition, Training, Sport, Stress

## Abstract

Practicing sport at the highest level is typically accompanied by several stressors and restrictions on personal life. Elite athletes’ lifestyle delivers a significant challenge to sleep, due to both the physiological and psychological demands, and the training and competition schedules. Inter-individual variability of sleep patterns (e.g., sleep requirements, chronotype) may have important implications not only for recovery and training schedules but also for the choice of measures to possibly improve sleep. This article provides a review of the current available literature regarding the variability of sleep among elite athletes and factors possibly responsible for this phenomenon. We also provide methodological approaches to better address the inter-individual variability of sleep in future studies with elite athletes. There is currently little scientific evidence supporting a specific influence of one particular type of sport on sleep; sleep disorders may be, however, more common in strength/power and contact sports. Sleep behavior may notably vary depending on the athlete’s typical daily schedule. The specificity of training and competition schedules possibly accounts for the single most influential factor leading to inconsistency in sleep among elite athletes (e.g., “social jet lag”). Additionally, athletes are affected by extensive exposure to electric light and evening use of electronic media devices. Therefore, the influence of ordinary sleep, poor sleep, and extended sleep as important additional contributors to training load should be studied. Future experimental studies on sleep and elite sport performance should systematically report the seasonal phase. Boarding conditions may provide a good option to standardize as many variables as possible without the inconvenience of laboratory. The use of interdisciplinary mixed-method approaches should be encouraged in future studies on sleep and elite sport. Finally, high inter- and intra-individual variability in the athletes’ sleep characteristics suggests a need for providing individual responses in addition to group means.

## Key points

- The sleep of elite athletes is affected by multiple sport-specific and societal factors
- Acute and chronic stressors affecting elite athletes may account for their greater measures of variability for sleep parameters than age- and sex-matched non-athlete controls
- Researchers need to consider methodological approaches that better account for the high inter- and intra-individual variability of sleep in elite athletes

## Review

Regular participation in physical activity induces many favorable health benefits including positive adaptations of the musculoskeletal, metabolic, and cardiovascular systems [1]. Sleep quantity and quality may also be improved by regular exercise. In a systematic review (21 studies; 16,549 participants in the age range 14–24 years), Lang et al. [2] found a positive relationship (effect size: *d* = 1.0) between the amount of physical activity and sleep. However, practicing a sport at the highest level offers a different set of circumstances, including high training load [3], high individual pressure with plenty of stressors, and constraints on personal life [4]. Elite sport poses a significant challenge to athlete’s sleep, due to the psychological and physiological demands as well as training/competition schedules which are prone to induce fatigue, states of hyperarousal, muscle soreness, and jet-lag symptoms [3, 5, 6]. Insomnia—defined by the cardinal symptoms of difficulty initiating or maintaining sleep (despite adequate opportunity to sleep), and/or non-restorative (unrefreshing) sleep, together with impaired daytime functioning [7]—is frequent among elite athletes, with sleep quality being most vulnerable prior to major competitive events, during periods of high-intensity training and following long-haul travel to competitions [8]. Unlike their non-athlete peers, athletes may face psychosocial stress about performance expectations from sports coaches, family, competitors, and even themselves [4, 9]. Finally, although the elite athlete population is often seen as the epitome of health, athletes are less prone to experiencing the beneficial effects of exercise on sleep when poor sleep quality exists [10]. Compromised sleep quantity and/or quality may be detrimental to the outcome of the recovery process after training and competition, which results in impaired muscle glycogen repletion, impaired muscle damage repair, alterations in cognitive function, and an increase in mental fatigue [11].

This article provides a review of the current available literature regarding (i) the variability of sleep among elite athletes and factors possibly responsible for this phenomenon; and (ii) methodological suggestions aimed to better address the inter-individual variability of sleep in future studies with elite athletes. An online literature search was performed using PubMed from inception of the database to March 2018 with the following keywords used in different combinations: “variability,” “variation,” “sleep,” “sleep loss,” “sleep deprivation,” “sleep restriction,” “sleep extension,” “performance,” “recovery,” “fatigue,” “glycogen,” “inflammation,” “immunity,” “injury,” “elite,” “athlete,” “sport,” “match,” “competition,” “training,” “stress,” “travel,” “light,” “nap,” “temperature,” “screen.” All titles and abstracts were carefully read and relevant articles were retrieved for review. In addition, the reference lists from both original and review articles retrieved were also reviewed.

Assessing the sleep quality and the prevalence of sleep disorders among a cohort of 107 professional ice hockey players, Tuomilehto et al. [12] found that one in every four players had a significant sleeping disorder (e.g., obstructive sleep apnea, restless legs syndrome and periodic leg movement, insomnia). Likewise, 25% of Canadian National Team athletes were identified to have clinically relevant sleep disturbances that required further clinical sleep assessment [13]. Relatively high levels of sleep complaints are prevalent among elite athletes, with a prevalence of sleep disturbance ranging from 13 to 70% (for a review, [8]). A systematic review revealed that the quality of research addressing sleep quality among elite athletes was low, with poorly designed studies and few controlled comparisons between athletes and non-athletes [8]. Moreover, it had been shown that Olympic athletes display lower sleep quality—including lower sleep efficiency and a higher fragmentation index—and that they have considerably larger measures of variability for sleep parameters than age- and sex-matched non-sporting controls [14]. Similarly, Whitworth-Turner et al. [15] showed that, despite longer sleep duration, youth soccer players engaged in a full-time academy displayed longer sleep latency, lower sleep efficiency, and higher intra-individual variability for both variables compared to non-athlete controls. Sleep duration and quality in elite athletes during training routine are depicted in Table 1. While it is difficult to separate the effects of sport and society on athletes’ sleep, we should acknowledge the influence that both can have on athletes’ performance [16].

**Table 1:** Sleep duration and quality in elite athletes measured using polysomnography, electroencephalogram system, actigraphy, or sleep logs

Study	Subjects	Age (years)	Sport(s)	Training and competition volume (h/week)	Time in bed (h:mins)	Sleep latency (mins)	Time asleep (h:mins)	Time awake/wake after sleep onset (h:mins)	Sleep efficiency (%)	Methodology
Fuller et al. [98]	21 M	22.5 ± 2.7	Australian Rules football, rugby			16 ± 16	7:27 ± 0:46	0:41 ± 0:22	88.7 ± 4.9	Polysomnography
Sargent et al. [117]	10 M	15.6 ± 0.5	Soccer		9:18 ± 0:54	18 ± 8	8:00 ± 0:42	1:04 ± 0:39	86.0 ± 5.0	Polysomnography
Taylor et al. [118]	7 F	19.0 ± 2.0	Swimming		7:50 ± 0:32	19 ± 4	7:31 ± 0:30	0:04 ± 0:06		Polysomnography
Tuomilehto et al. [12]^a^	23 M		Ice hockey		8:59	18	6:55	0:54		Polysomnography
Whitworth-Turner et al. [15]	12 M	19.0 ± 1.0	Soccer		9:00	25 ± 9	8:06 ± 0:33	0:12	93.0 ± 3.0	EEG system
Caia et al. [80]	7 M	24.3 ± 2.1	Rugby		8:00 ± 0:30	17 ± 8	6:54 ± 0:24		87.3 ± 3.0	Actigraphy
Caia et al. [102]	15 M	25.5 ± 3.7	Rugby		8:35 ± 1:02	16 ± 18	7:31 ± 0:55		87.8 ± 3.8	Actigraphy
Carriço et al. [119]	25 M	26.3 ± 4.7	Soccer		7:40 ± 0:42	24 ± 9	6:36 ± 0:45	0:30 ± 0:16	85.0 ± 5.0	Actigraphy
Dunican et al. [92]	9 M/F	18.9. ± 2.9	Judo			26 ± 36	7:25 ± 0:07	0:06 ± 0:04	89.0 ± 6.0	Actigraphy
Eagles and Lovell [120]	10 M	24.3 ± 2.6	Rugby				7:41 ± 1:29		87.0 ± 2.0	Actigraphy
Knufinke et al. [44]	98 M/F	18.8 ± 3.0	Road cycling, triathlon, mountain bike, handball, volleyball, soccer	19.3 ± 5.1	8:32 ± 1:10	14 ± 16	7:50 ± 1:08	0:33 ± 0:17	88.5 ± 5.5	Actigraphy
Lastella et al. [26]	124 M/F	22.2 ± 3.0	Australian Rules football, basketball, soccer, rugby, cycling, mountain bike, race walking, swimming, triathlon		8:24	19	6:48		86.2	Actigraphy
Lastella et al. [121]	21 M	19.9 ± 1.7	Cycling		9:12 ± 0:18	28 ± 14	7:24 ± 0:36		86.4 ± 0.4	Actigraphy
Leeder et al. [14]	46 M/F		Canoeing, diving, rowing, short track speed skating		8:36 ± 0:53	18 ± 17	6:55 ± 0:43	1:17 ± 0:31	80.6 ± 6.4	Actigraphy
Mah et al. [29]	11 M	19.4 ± 1.4	Basketball				6:41 ± 1:02			Actigraphy
O’Donnell et al. [122]	26 F	23.0 ± 6.0	Netball		9:05 ± 0:47	29 ± 16	7:16 ± 0:51		80.6 ± 6.5	Actigraphy
Pitchford et al. [123]	19 M	22.1 ± 3.5	Australian Rules football		8:17 ± 0:32		6:59 ± 0:26	1:09 ± 0:30	84.7 ± 6.5	Actigraphy
Richmond et al. [124]	19 M	24.1 ± 3.3	Australian Rules football				8:51 ± 0:06	0:42 ± 0:06	92.5 ± 1.3	Actigraphy
Robey et al. [125]	12 M	18.5 ± 1.4	Soccer			21 ± 11	7:13 ± 0:39	0:15 ± 0:11	89.4 ± 5.8	Actigraphy
Sargent et al. [64]^b^	7 M/F	22.5 ± 1.7	Swimming		7:42 ± 0:54	41 ± 43	5:24 ± 1:18		70.7 ± 15.1	Actigraphy
Sargent et al. [68]	70 M/F	20.3 ± 2.9	Australian Rules football, basketball, mountain bike, race walking, road cycling, swimming, triathlon		8:18 ± 1:18		6:30 ± 1:24		85.6 ± 7.2	Actigraphy
Schaal et al. [126]	10 F	20.4 ± 0.4	Synchronized swimming		8:32 ± 0:13	17 ± 2	7:13 ± 0:11		84.7 ± 1.3	Actigraphy
Shearer et al. [127]	28 M	24.4 ± 2.9	Rugby		8:49 ± 0:49	34 ± 40	7:04 ± 1:01	1:05 ± 0:39	79.0 ± 9.2	Actigraphy
Suppiah et al. [27]^b^	11 M	14.8 ± 0.9	Badminton, bowling		6:58	5 ± 8	6:07 ± 0:39	0:38 ± 0:16		Actigraphy
Suppiah et al. [17]^b^	29 M	14.7 ± 1.3	Track and field sprint, shooting		6:45 ± 0:29		5:28 ± 0:33	1:15 ± 0:23	80.9 ± 8.6	Actigraphy
Thornton et al. [85]	31 M	24.5 ± 3.9	Rugby		8:16 ± 1:13	21 ± 19	7:17 ± 01:07	0:42 ± 0:17	88.1 ± 4.2	Actigraphy
Thornton et al. [45]	14 M	26.1 ± 2.9	Rugby		7:29 ± 1:21		6:55 ± 1:14	0:35 ± 0:16	92.3 ± 3.0	Actigraphy
Van Ryswyk et al. [91]	19 M	23.7 ± 2.0	Australian Rules football		8:35 ± 0:48		7:07 ± 0:55		82.3 ± 6.5	Actigraphy
Miller et al. [46]	16 M		Australian Rules football		8:29 ± 1:19	18 ± 22	6:49 ± 1:13	1:10 ± 0:33	85.5 ± 5.7	Actigraphy
Miller et al. [46]	7 M		Soccer		8:02 ± 2:11	10 ± 15	6:42 ± 2:00	0:57 ± 0:24	85.5 ± 5.7	Actigraphy
Miller et al. [46]	28 M		Rugby		8:25 ± 1:41	9 ± 11	7:10 ± 1:34	0:56 ± 0:23	88.5 ± 4.2	Actigraphy
Dumortier et al. [3]	26 F	15.4 ± 3.7	Artistic gymnastics	25.0 to 32.3	9:44 ± 0:42	28 ± 12	9:03 ± 0:46	0:05 ± 0:05	93.0 ± 3.7	Sleep logs
Fullagar et al. [128]	15 M	25.5 ± 4.9	Soccer			20 ± 17	8:32 ± 1:11		91.6 ± 3.7	Sleep logs
Fullagar et al. [96]^b^	16 M	25.9 ± 7.5	Soccer			16 ± 7	8:44 ± 0:40	0:22 ± 0:39		Sleep logs
Knufinke et al. [89]	98 M/F	18.8 ± 3.0	Road cycling, triathlon, mountain bike, handball, volleyball, soccer	19.3 ± 5.1		20 ± 14	8:11 ± 0:44	0:13 ± 0:19		Sleep logs

Values are means ± standard deviation

*EEG* Electroencephalogram, *F* Female, *M* Male

^a^Suspected sleep disorder based on questionnaire

^b^Week days or training days

### Factors Potentially Accounting for Sleep Variability Among Elite Athletes

Sleep is affected by many factors, e.g., training, competition, travel, wake-length prior to sleep, regularity of sleep-wake schedules (social jet lag), sleeping environment, and light exposure [8, 17]. While it is difficult to separate the effects of sport and society, we should be aware of the influence that both can have on athletes’ sleep [16]. In this section, we explore the influence of sport-specific factors—type of sport, training load, chronotype, training/competition time, jet lag, seasonal phases—as well as environmental (sleep environment and electronic media devices) and methodological factors on sleep.

#### Type of Sport

Sleep comprises distinct stages: non rapid eye movement-sleep (NREM), divided into stages 1, 2, 3, and rapid eye movement (REM) stage. During sleep, metabolic activity is at its lowest point (i.e., slow breathing, low heart rate, and low cerebral blood flow) and the endocrine system increases the secretion of growth hormone via the pituitary gland allowing physiological restitution [18, 19]. Learning and the consolidation of motor memory are associated with slow-wave sleep (i.e., NREM stage 3), REM sleep [20–22], and sleep spindles [23] during the night, and result in an overnight plastic reorganization on a systemic level within the brain, including increased activation of the primary motor cortex [24]. There is currently little scientific evidence to support a specific influence of a given type of sport on sleep. Brandt et al. [25] described the perceived sleep quality of elite athletes during a competitive period, and found no significant differences between sports modalities (individual versus team sport). Conversely, it had been shown previously that athletes from individual sports go to bed earlier, wake up earlier, and obtain less sleep (individual vs team; 6.5 vs 7.0 h) than athletes from team sports [26]. Sleep disorders are also more prevalent in esthetic sports (33%), while athletes in high risk sports (i.e., sliding, aerial, and motor sports) had significantly less sleep issues than the others [4].

From an acute perspective, Suppiah et al. [27] showed marked differences in sleep patterns between high-intensity athletes (i.e., badminton players), who displayed significantly greater amounts of slow-wave sleep, less light sleep, and better sleep continuity than low-intensity athletes (i.e., bowlers). Conversely, the same authors group [17] showed no differences in electroencephalographic obtained sleep stages between student-athletes participating in high- (i.e., track and field) and low- (i.e., shooting) intensity sports, following a day of sport-specific training. Increased exercise intensity may elevate nocturnal heart rate compared to a day without exercise, but it has no effect on nocturnal heart rate variability, actigraphic, or subjective sleep quality [28]. Additionally, the diversity among the included types of sport, differences in training loads, and light exposure during practice, together with the small number of participants, may account for discrepancies between results. Future studies are required to assess the hypothetic influence of specific types of sport on sleep. In this respect, the reciprocal influence of REM sleep and the vestibular system that controls balance and body position during sport skills is particularly appealing [29–31]. From a chronic perspective (i.e., 6-day microcyle with two training sessions per day), sleep parameters of participants training in strength sport remained constant over the entire process, whereas the high-intensity interval training group experienced unfavorable sleep tendencies during the training phase (e.g., lower objective sleep efficiency). Consequently, it seems that high-intensity interval training protocols have a stronger short-term effect on sleep and need for recovery than strength training [32]. Independent of training content, regular exercise and training may also increase the amount of slow-wave sleep, even in a no-exercise condition [33]. Consequently, the overall fitness of subjects may be a potential moderator variable to consider [33].

Sleep disorders may be more common in specific types of sport than others [34]. The prevalence of obstructive sleep apnea appears to be much higher in strength power athletes (e.g., rugby) than the general population, possibly due to a large body mass and neck circumference [35, 36]. In collision sport athletes, a high body mass index (> 28 kg/m^2^) and large neck circumference (> 40 cm) are considered performance-enhancing assets [36]. However, these same physical traits also increase an individual’s risk of obstructive sleep apnea [35, 37]. Furthermore, the incidence of obstructive sleep apnea appears to be higher again in American Football players with these physical traits [38, 39]. Moreover, athletes practicing contact sports who experienced concussions during the previous year reported more symptoms of sleep disturbance and poorer sleep quality than a control group of athletes without a history of concussion, matched for age, sex, education level, and age at which they started playing organized sports [12, 40], whereas subjects with a low sleep quantity the night before the concussion reported both a greater number of symptoms and more severe symptoms after the concussion [41].

#### Training

Several studies have assessed the influence of training load on sleep. Kölling et al. [42] investigated the sleep-wake patterns of 55 junior (17.7 ± 0.6 years) national rowers via sleep logs and actigraphy during a 4-week training camp. Objective sleep measures revealed less total sleep time in the first 2 weeks, while training volume and intensity were higher. In the second half of the camp, fewer training sessions were held, more afternoons were training free, and total sleep time was longer. Hague et al. [43] have shown that, with a reduced exercise load, slow-wave sleep pressure may be reduced, resulting in lower levels of slow-wave sleep (15.5 min) and increased rapid eye movement sleep (17.7 min) among subjects who perform aerobic type exercise daily (15.1 ± 4.0 h/week). Dumortier et al. [3] found that high training loads lead to lower total sleep time—as assessed with sleep logs—the following night, while lower total sleep time also causes athletes to perceive the training load as higher the following day, among elite female artistic gymnasts. However, Knufinke et al. [44] showed that day-to-day variation in training load had neither a significant effect on sleep quantity nor significantly affected sleep stage distribution among a cohort of 98 elite athletes. The experimental design, the performance level of the participants, the wide range of sporting backgrounds, and differences in training load may explain discrepancies between results. In addition to training load, specific physical demands encountered during training may potentially alter sleep. Thornton et al. [45] investigated the influence of changes in training loads on subsequent night-time sleep. Results showed that increased acceleration/deceleration load was related to a higher sleep efficiency. Although no objective or subjective measures of fatigue were collected in this study, these results may suggest that training that involves greater acceleration/deceleration efforts may exacerbate athletes’ perceptions of fatigue, resulting in athletes attempting to increase their quantity of sleep [45]. Miller et al. [46] found that Australian Rules footballers experienced more sleep disturbances, compared to rugby union players and soccer players, possibly because of the combination of high aerobic demand and high physical contact leading to pain and movements during sleep. However, the influence of exercise-induced delayed onset muscle soreness (DOMS) on sleep quantity/quality is still debated [47, 48].

Injuries too may disrupt usual sleeping patterns. During the return period from injury, a player is usually involved in the gym and/or treatment room and/or on the pitch several times per day which may prevent napping [49]. For the elite athlete, daytime napping not only serves as an effective recovery tool between training sessions which occur on the same day, but is also used as a strategy to extend sleep. Napping behavior for individual and team sports athletes was documented by Lastella et al. [26]. Nap frequency (i.e., the percentage of days on which a nap is taken) was 15 and 11% among individual and team sport athletes, respectively [26].

Several training strategies are increasingly used by athletes in an attempt to enhance training stress and thus physiological adaptations [50]. Louis et al. [51] assessed the influence of a “sleep low” strategy, consisting of sleeping with reduced glycogen availability, on performance and sleep patterns among trained triathletes. Results showed that the sleep low group (i.e., high-intensity training sessions in the evening followed by overnight fast and performing a subsequent low-intensity training session in the morning) significantly improved 10 km running performance (*P* < 0.01, *d* = 0.38), whereas no improvement was recorded in the control group (i.e., maintained glycogen availability during the overnight recovery period). Additionally, in the sleep low group, sleep efficiency decreased slightly, by 1.1% (*P* < 0.05, *d* = 0.25), compared to the control group. The live high-train low (LHTL) training strategy, where athletes sleep at moderate altitude while training at lower altitudes or near sea level, is also used successfully by many endurance athletes. However, Saugy et al. [52] found that sleeping is altered more in moderate hypobaric hypoxia than in normobaric hypoxia during a LHTL altitude camp, even if no difference in the 3-km run time improvement was found between conditions.

#### “Chronobehavior”: Chronotype and Training/Competition Time

Chronotype is a genetically determined predisposition that modifies each individual’s preference to be most active in the morning (morning-type), middle of the day (neither-type), or in the evening (evening-type) [53, 54]. In elite sport, extreme chronotypes (i.e., definite morning type or definite evening type) are unusual and chronotype distribution mainly depends on the chosen sport and respective training time of the athletes [55–57]. Vitale et al. [58] showed that sleep quality—moving time, immobility time, sleep efficiency, actual sleep time—was poorer in the morning-type than in the evening-type players after the evening high-intensity training session (20:00), whereas no significant changes to the sleep quality of the two chronotypes was observed after the morning session (08:00). However, a limitation of this study is the fact that morning-types generally have consistently early bedtimes and waking times. In this respect, the reduction in actual sleep time in the morning-type players after the evening training session may be due to the chronotype rather than to the evening training session itself. Sleep behavior may consequently vary depending on the athlete’s chronotype and the time of day an athlete is required to train/compete [58] or work. Subsequent sensitivity of physical performance to sleep loss may be inter-individually variable and may relate to chronotype, usual bedtimes or sleep lengths [59]. For example, “night owls” who prefer to go to bed later and sleep in (e.g., 1:00–9:00) and who then have to wake up at 7:00 to train at 9:00 will cut their sleep by several hours per night, missing critical periods of sleep [55]. As resting periods and sleep-wake schedules are normally planned for the entire training group/team regardless of individual distinctions, planning individualized schedules based on athletes’ preferred sleep schedules may be an effective measure to restore good sleep [55, 60]. It is proposed that there is a relationship between circadian rhythms and athletic performance with peak athletic performances at a specific time of day [61]. However, exercise training altered the diurnal variations of performance with greater improvement in performance at the time of day at which training is conducted [62]. In this respect, Rae et al. [63] showed that grouping swimmers by chronotype and usual training time allowed them to detect significant diurnal variation in performance, such that morning-type swimmers and those who usually train in the morning were faster in the 06 h30 time trial than the neither-type swimmers and those habitually training in the evening, respectively. In addition to chronotype, the influence of training/competition time is crucial to consider. For example, the common practice of early morning training (i.e., before 8 am) in many sports (e.g., swimming) may be associated with a reduction in total sleep, higher daytime sleepiness, and poorer sleep quality compared to rest days [64] or training during the day [36]. In this respect, altering the athletes’ academic and training schedules around an extended sleeping period is vital to increase sleep time, especially for student athletes [15].

#### Jet Lag

Social jetlag is the discrepancy—particularly evident among evening chronotypes—between circadian and social clocks, which is measured as the difference in hours in midpoint of sleep between work days and free days [65]. Adolescents’ sleep patterns undergo a phase delay, that is, a tendency toward later times, for both falling asleep (11:00 pm or later) and waking if left undisturbed, and display irregular sleep-wake patterns including significant discrepancies between weekdays and weekends [3, 66]. These biological and behavioral factors, together with the “forbidden zone for sleep”—the early evening period when it can be difficult to initiate or maintain sleep even if one is in bed—make it difficult to encourage adolescent athletes to go to bed earlier and increase their sleep duration in this way [67]. Allowing athletes to sleep longer the next morning might be a more productive strategy to increase sleep duration [68] as long as sleep quality is maintained. Adjusting to an early school/training schedule following weekend or vacation periods during which very late schedules are typically kept can take several days to several weeks, resulting in excessive sleepiness during the day [69]. While variability is a characteristic component of sleep-wake behavior [70], regularly heightened levels of inconsistency in sleep are considered unfavorable and thought to disrupt the synchrony of circadian rhythms, subsequently influencing sleep duration/quality, and performance [71, 72]. However, the specificity of training and competition schedules is prone to inducing inconsistency in sleep among elite athletes [3]. For example, an athlete may continue sleeping on the morning of a day off, before waking up early on a training day [64]. Elite athletes performing at night can be compared to night shift workers who are required to perform at their peak at a time that is incongruent to their circadian rhythm [11]. Travel fatigue resulting from a domestic flight is a complex summation of physiological, psychological, and environmental factors that accrue during an individual trip, such as prolonged exposure to mild hypoxia and cramped conditions with restricted activity [73, 74]. Several studies have assessed the acute effects of short-haul air travel without crossing time zones (total travel time between 3 and 5 h) on performance and perceptual measures among team sport players [73, 75, 76]. Overall, results showed that travel has no effect on indicators of performance but negatively influences perceptual measures (e.g., reduced alertness, greater stress). It must be noted that travel fatigue could be cumulative and may accrue over the course of a season depending on the distances traveled, frequency of trips, and length of the season [74]. The negative effects of 21-h-long international travel across seven times-zones on sleep have also been documented [77]. Specifically, travel east has a greater detrimental effect on sleep (i.e., later sleep onset and sleep duration reduction), subjective jet-lag, fatigue, and motivation compared to travel west, among physically trained participants [77]. Elite athletes who join their national team for international competitions or travel to pre-season training camps may be consequently exposed to the negative effects of travel across multiple time zones on sleep [78].

#### Seasonal Phases

Sleeping patterns may vary throughout the season, depending on the training/competition period, e.g., pre-season, one-day or multi-day competition and off-season phases. Swinbourne et al. [36] showed that highly trained team-sport athletes report longer sleep times during the off-season (8.2 ± 1.1 h) compared to athletes during the pre-season or in-competition (˂ 8 h) phases. Accordingly, Pittsburgh Sleep Quality Index (PSQI) scores are best during the off-season and poorest during the competition phase, whereas athletes appear to be sleepier during the day in the pre-season training phase, which may be a result of inadequate sleep, and/or a greater sleep requirement due to training loads and intensity [36]. However, it is important to note that the PSQI has not been validated in an athlete population. In this respect, the Athlete Sleep Screening Questionnaire was developed as a sleep screening tool for detecting clinically significant sleep disturbances and daytime dysfunction, and to provide interventions based on the type and severity of the problem detected within an athlete population [13, 79]. Differences in bedtime and wake time during pre-season may also be observed when compared to the competitive season, both for players and staff [80]. Multi-day competitions may additionally exert chronodisruptive effects on sleep, especially when late evening and/or early morning competitions are scheduled [71, 81]. Depending on the seasonal phase, different levels of perceived stress may account for these results. Alterations in perceived stress and sleep quality have actually been reported after the more intense periods of training and competition among English elite football players [82]. Athletes may also experience disturbed sleep prior to important competition or games with problems falling asleep due to thoughts about competition and nervousness [83]. Moreover, seasonal phases generally correspond to specific environmental conditions. In this respect, future experimental studies on sleep and elite sport should systematically report seasonal phase and, ideally, be conducted during the early fall or winter to minimize the effects of environmental conditions, i.e., outdoor light levels [84] and morning/afternoon temperature differences [57].

#### Sleep Environment

Professional athletes often spend nights in an unfamiliar hotel environment throughout the season, before home and away matches [49] and during training camps [85]. Described by Suetsugi et al. [86], “the first night effect” may potentially contribute to sleep impairment in such circumstances. The first-night effect is thought to result from an individual’s lack of adaptation to the unfamiliar environment (usually a sleep laboratory) [86]. The main characteristics of this effect are decreased total sleep time, decreased REM sleep, and a lower sleep efficiency index [86]. Thornton et al. [85] investigated the effects of a training camp on the sleep characteristics of professional rugby league players compared to a home period. During the training camp, total sleep time (− 85 min), time in bed (− 53 min), and sleep efficiency (− 8%) were reduced when compared to home. Factors such as noises in and/or outside a room [87] and altered schedules while away compared with home [76] may be identified as additional reasons for poor sleep during training camps.

#### Electronic Media Devices

Sleep duration has declined over the last centuries mainly because of the altered exposure to the natural light-dark cycle, including the wide availability of electric light, reduced exposure to daylight within buildings, and evening use of light-emitting devices [88]. Athletes do not escape the impact of electronic media devices. Investigating the sleep hygiene practices of elite (youth) athletes aged 18.8 ± 3.0 years, Knufinke et al. [89] found that athletes engaged in sedentary (blue-light emitting) activities within the last hour before bedtime in 70% of the monitored nights. Anecdotally, many athletes view sleep as a flexible commodity that can be exchanged for activities that are viewed as either more important or more enjoyable [16]. Bedtime procrastination is defined as failing to go to bed at the intended time, while no external circumstances prevent a person from doing so, i.e., the person has no actual constraints keeping them from going to bed on time, but “just” fails to do so [90]. Kroese et al. [90] speculate that it is not so much a matter of not wanting to sleep, but rather of not wanting to stop doing other activities (e.g., electronic media devices consultation). Nevertheless, electronic devices may be useful to deliver a sleep optimisation programme. Van Ryswyk et al. [91] used electronic devices to deliver ongoing educational feedback in the form of weekly group electronic messages (email and text message) to keep the athletes engaged in the intervention. Results showed a significant increase in total sleep time and sleep efficiency, as measured by sleep diaries, but no significant difference in total sleep time using actigraphy [91]. Likewise, Dunican et al. [92] demonstrated the difficulty in increasing sleep duration by providing an environment more conducive to sleep in the evening. Removing electronic devices on two consecutive nights resulted in no significant within-group or between-group differences in any sleep measure among elite judo athletes. Future studies are required to distinguish the potential negative effect of blue-light exposure on sleep and the arousal-reducing nature of the accompanying activity (e.g., watching a movie, engaging in social media through the use of orange-tinted glasses) [89]. As excessive sleepiness is associated with reduced short-term memory, learning ability, and behavioral control [93], the success of educational efforts may rely on athletes having achieved sufficient sleep before the start of the educational programme. Additionally, many athletes like to keep their phone accessible at night to ensure communication can be maintained. This “fear of missing out” and expectation of potential interaction with others can result in an “on-call” effect. This effect is best demonstrated in workers who are “on-call” and often report poor sleep due to anticipatory effects [16, 94]. Sleep continuity disruption is especially harmful for slow-wave sleep and positive mood, compared to restricted sleep time [95]. Sleep maintenance difficulty is the most commonly reported symptom of insomnia and is a common feature of sleep in a variety of other contexts, such as early parenthood [70, 95, 96].

#### Actigraphy Settings

Actigraphy has emerged as a valid alternative to polysomnography for collecting objective sleep data simultaneously with multiple elite athletes over consecutive nights [92, 97]. However, choosing the correct sleep-wake threshold is a methodological issue that may influence athletes’ sleep results. The incidence of poor sleep among athletes may even become more pronounced in case of default application of actigraphy settings [44]. Thresholds that have a high sensitivity to sleep (i.e., above 80 activity counts is scored as being awake) yield the best combination of agreement, sensitivity, and specificity among endurance-trained cyclists assessed during a 6-week block of heavy training [97]. However, medium sleep-wake thresholds (activity counts above 40) should be used to process sleep data for elite team sport athletes [98] in pre-season training. The differences between the findings of these two studies conducted on athletes may relate to the different subject groups and conditions in which studies were conducted. As previously discussed, heavy training loads may reduce the immobility of subjects during sleep, with the possibility that cyclists moved more in their sleep due to muscle soreness induced by their training [97, 98]. Future studies using actigraphy should systematically indicate which sleep-wake threshold is used to process the data.

#### Summary

Several factors could account for the variability of sleep among elite athletes. While there is currently little scientific evidence supporting a specific influence of one particular type of sport on sleep, sleep disorders may be more common in strength/power and contact sports. The specificity of training and competition schedules is possibly the single most influential factor leading to inconsistency in sleep among elite athletes. The influence of ordinary sleep, poor sleep, and extended sleep as important additional contributors to training load should be studied.

### Methodological Considerations

As previously mentioned, sleep is influenced by numerous factors that have the potential to affect athletes’ sleep variables. Consequently, researchers need to take these factors into account, from the planning of scientific research to the presentation of results. In the present section, we propose methodological approaches that better account for the inter-individual variability of sleep in future studies with elite athletes.

#### Characterization of Athletes

Few guidelines are currently available to help researchers define “expertise” or “eliteness” as objectively as possible in the study of sport. Such imprecision in the criteria used to define participants as “elite” athletes threatens the validity of research on expertise in sport and sleep, especially given elite athletes’ unique lifestyle. As many variables as necessary have to be reported to describe the participants level of performance and training/competition schedule appropriately. Swann et al. [99] proposed a taxonomy for classifying the validity of expert samples. The following equation was proposed: “Eliteness”/expertise of athletic sample = [(A + B + C/2)/3] x [(D + E)/2]. The taxonomy ranks participants on a continuum (score range 1–16) depending on five variables—i.e., athlete’s highest standard of performance (A), success at the athlete’s highest level (B), experience at the athlete’s highest level (C), competitiveness of sport in athlete’s country (D), and global competitiveness of sport (E)—and should be used in future studies on sleep in elite athletes [13, 91]. The influence of age on sleep is well documented, both in the general [100] and athlete populations [3, 36]. Age may also be a relevant factor to take into consideration in terms of the effects of sleep and circadian rhythm on athletic performance as older subjects are generally more morning oriented [101]. Age and corresponding category may have a profound effect on the athletes’ sleep schedules (i.e., bed and wake time) with a potentially harmful transition from junior delayed sleep-wake patterns to senior early morning training requirements [102]. The degree of familiarization with elite athletes’ unique lifestyle may possibly influence sleep outcomes when dealing with jet lag, unfamiliar sleep environment, and/or sleep disturbance before and after important competitions.

#### Mixed-Method Approach

A promising approach could be to view sleep as a manifestation of the metaregulation process [103]. According to this view, sleep is not a distinct entity, which is regulated independently and has a specific function, but rather represents the process of metaregulation, which reflects an interaction between internal and external factors (e.g., light, ambient temperature), preceding waking activities and current homeostatic needs [103]. In this respect, the use of mixed-method approaches should be encouraged in future studies on sleep and elite sport. A mixed-method approach is an interdisciplinary approach combining objective assessment with qualitative interviews, and has already been used in nutrition [104] and sleep [49] studies. As qualitative methods are able to investigate why athletes cannot achieve an adequate sleep for performance and recovery, future studies should combine quantitative and qualitative methods to investigate not only how much athletes sleep but also how cultural and psychological factors affect their sleep.

#### Individualized Approach

Research examining the sleep of athletes has typically averaged data across several nights, providing a mean estimate of usual sleep [14, 26, 36]. While such approaches are useful to allow basic insight into sleep, they lack the sophistication to provide an understanding of how sleep may vary across multiple nights [70]. Intra-individual variability reflects differences within individuals over time [105] and has been used to examine the extent to which sleep varies with age [102] and activity level [15]. High inter- and intra-individual variability in the athletes’ sleep characteristics indicates the need for individualized sleep education strategies and interventions to promote appropriate sleep [106, 107]. In this respect, it may be encouraged for future research to include the presentation of sleep data encompassing individual responses, in addition to group means [107–109]. Moreover, a Bayesian approach comparable to that developed for the athlete’s biological passport has been recently proposed to individualize monitoring of muscle recovery in athletes [110]. The procedure starts with a sport-specific prior distribution and reference ranges based on measurements from an independent sample of athletes, to be used as starting points for the individualization procedure. It then successively integrates information from individual measurements, in order to progress toward individualized distributions for recovered and non-recovered values. Reference ranges are based on cross-sectional data and therefore include inter- as well as intra-individual variation. Future studies are required to assess if such an approach may be suitable for the variability issue of sleep.

#### Field-Based Studies Versus Laboratory Studies

Another parameter to take into account for studies on sleep and elite sport concerns the balance between field-based and laboratory studies. Tracking sleep in the field appears to be valid but is limited in practice. It is virtually impossible to take all influencing factors into consideration when studying a homogenous group of elite athletes [111]. In terms of implementation, there is a need to take into account the broad ecological context of the sporting environment, including identifying intrapersonal factors, sociocultural factors, policies, and the physical environment [112]. Tracking sleep in a laboratory setting can be interesting to both control and manipulate some variables. However, the applicability of findings arising from a laboratory setting can be questioned in relation to the actual sport context [113]. It is impractical to apply the methodology used in a sleep laboratory, i.e., polysomnography, on a daily basis. The application of electrodes before going to bed requires a long period of time, at least 30 min. Thus, the detrimental effect of late competition on bedtime and sleep duration [49, 60, 96] may be even more pronounced when polysomnography procedures are used. Boarding arrangements [3, 17] may represent a compromise to standardize as many variables as possible without the inconvenience of a laboratory, e.g., time and type of food ingestion, light exposure, temperature [114], behaviors in the proximate period to bedtime [115], and regularity of sleep-wake schedules. Concerning this latter point, training and school schedules which reduce total sleep time can also have a significant impact on sleep structure, especially among insomniacs. The athlete should get up in the morning at the same time even if he/she experienced low-quality sleep the previous night, in order to increase sleep need, reduce sleep onset latencies, increase sleep efficiency, and establish consistent sleep and wake-up times [116].

#### Summary

Methodological suggestions aimed to better address the high inter- and intra-individual variability of sleep in elite athletes notably include boarding conditions, interdisciplinary mixed-method approaches, and the presentation of individual responses in addition to group means.

## Conclusions

The sleep of elite athletes is influenced by many factors, both societal (e.g., electronic media devices) and sport-specific factors (e.g., training, seasonal phases, competition, travel). All these acute and chronic stressors experienced by elite athletes may partly account for large inter- and intra-individual sleep variabilities. Sleep behavior is related to a complex summation of physiologic, psychologic, and environmental factors, and it is virtually impossible to take all of them into consideration. New methodological approaches are consequently required to increase the quality of the research addressing sleep among elite athletes. In this respect, the use of mixed-methods and interdisciplinary approaches combining objective assessment with qualitative data should be encouraged in future studies on sleep and elite sport.

## Abbreviations

DOMS: Delayed onset muscle soreness
LHTL: Live high-train low (altitude camp)
PSQI: Pittsburgh sleep quality index (questionnaire score)
REM: Rapid eye movement (sleep stage)
